# First report on identification of *Salmonella* Abortusovis from ovine abortion cases in Kazakhstan

**DOI:** 10.3389/fvets.2025.1717314

**Published:** 2026-01-19

**Authors:** Assiya Mussayeva, Zhandos Abay, Aigul Dossanova, Raikhan Nissanova, Merey Yerishov, Natalya Yegorova, Altynay Arysbekova, Malik Yussupov, Perizat Akshalova, Vladimir Kirpichenko, Yergali Abduraimov, Kunsulu Zakarya, Aralbek Rsaliyev, Ainur Nurpeisova, Markhabat Kassenov

**Affiliations:** 1 Kazakh Scientific Research Veterinary Institute LLP, Almaty, Kazakhstan; 2 Kazakh Medical University of Continuous Education, Almaty, Kazakhstan; 3 JSC National Holding Qazbiopharm, Astana, Kazakhstan

**Keywords:** *Salmonella enterica* serovar Abortusovis, ovine salmonellosis, molecular diagnostics, 16S rRNA, Kazakhstan

## Abstract

*Salmonella enterica* subsp. *enterica* serovar *Abortusovis* is a host-adapted ovine pathogen responsible for late-term abortions and significant economic losses in sheep farming. Despite its recognized importance, up-to-date epizootic data on the global distribution and genetic diversity of this serovar remain notably scarce. In this brief communication, we report the isolation and 16S rRNA-based identification of six *S.* Abortusovis isolates recovered from five aborted lambs during a single outbreak in the Karaganda region. Bacterial isolation was performed using non-selective enrichment on meat-peptone broth and plating on meat-peptone agar, followed by 16S rRNA gene amplification yielding ~1,100 bp PCR products. Six isolates were identified by 16S rRNA sequencing, showing 99% identity with *S. enterica* serovars Choleraesuis and Paratyphi C. Phylogenetic analysis confirmed their taxonomic position. Phylogenetic analysis using the Maximum Likelihood method (MEGA 11) placed the isolates within a clade including serovars Choleraesuis and Paratyphi C, suggesting close evolutionary proximity. Detailed analysis of electropherograms confirmed the purity of cultures and excluded contamination. Given the lack of recent molecular surveillance data on *S.* Abortusovis from many regions, including Central Asia, our findings fill an important gap and provide reference material for comparative studies. Further molecular monitoring is needed to support robust diagnostics, trace transmission pathways, and inform regional control strategies against ovine salmonellosis.

## 1 Introduction

Ovine salmonellosis, caused by *Salmonella* Abortusovis (*S.* Abortusovis), is an infectious disease leading to late-term abortions, stillbirths, and the birth of weak lambs (1). It is associated with endometritis in ewes, reduced fertility, and significant economic losses in sheep farming. Outbreaks can occur even in previously unaffected regions, as illustrated by the 2005 epidemic in Switzerland (2).

The etiological agent, *S.* Abortusovis, is a Gram-negative bacterium of the *Enterobacteriaceae* family with high environmental resistance (3, 4). It primarily affects the reproductive system of sheep, and susceptibility to infection depends on physiological status, immune competence, and stress factors (5–7).

**Figure 1:**
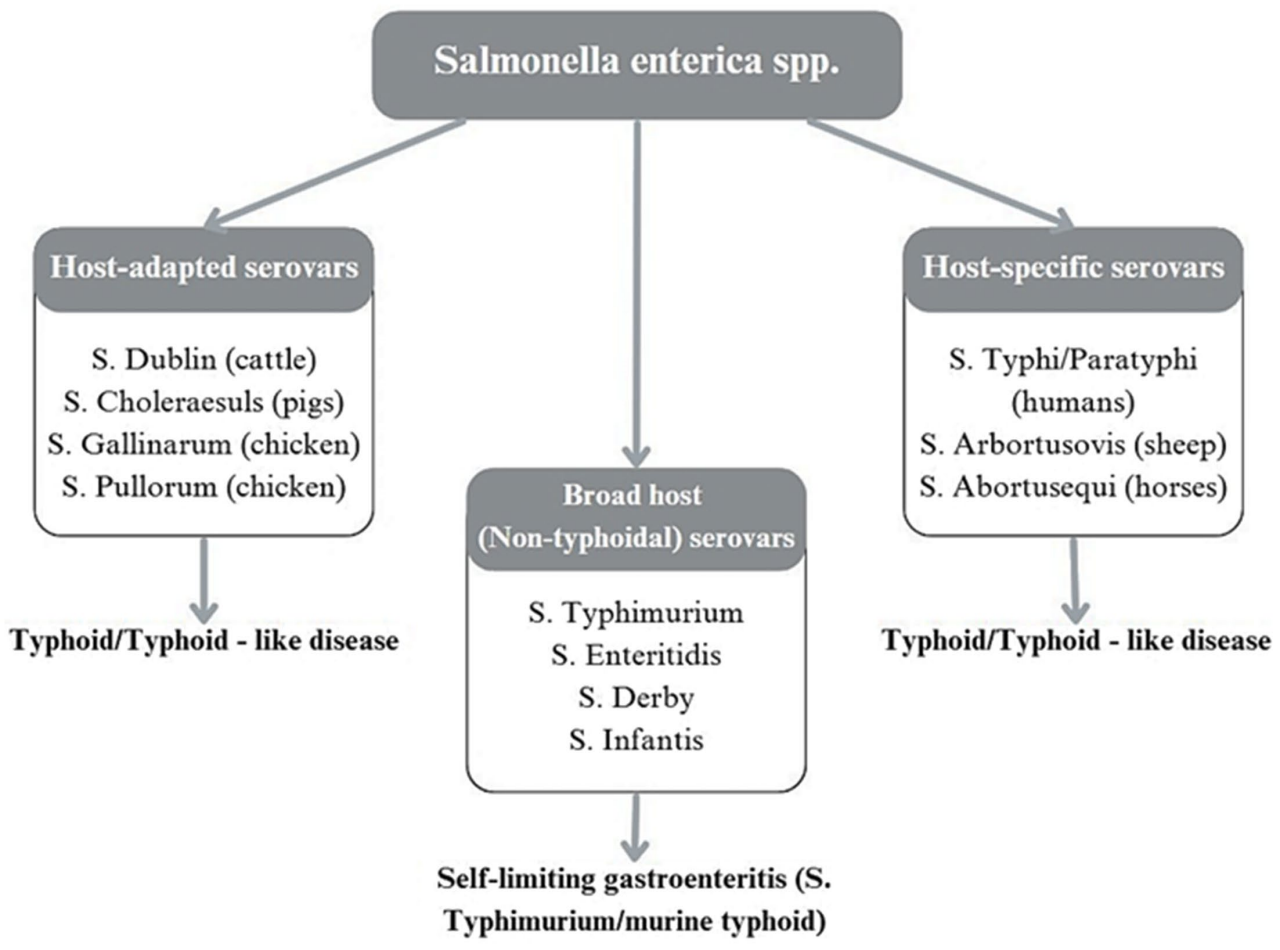
Classification of *Salmonella enterica* serovars based on host interaction and disease manifestation.

Understanding *S.* Abortusovis is essential to study its adaptation to hosts and genetic determinants of virulence (8). As a sheep-specific pathogen, *S.* Abortusovis is classified among host-restricted serovars (Figure 1) (9).

Transmission occurs mainly via the oral route, but vertical transmission from ewe to offspring also plays an important role (10, 11).

Factors such as overcrowding, poor hygiene, and immunodeficiency favor its spread, while asymptomatic carriers maintain persistent epizootiological risks (12, 13).

The global epizootiological situation is heterogeneous: Europe reports most cases due to active monitoring (8, 14–17), while information from Asia remains limited, with only a few studies documenting its occurrence (18). Data on *S.* Abortusovis in Central Asia are particularly scarce, and to our knowledge, no peer-reviewed reports describe its isolation or molecular characterization in the region. No peer-reviewed publications have been identified on the occurrence or molecular detection of *S.* Abortusovis in sheep in Russia or China. This absence of data from the two largest neighboring countries further emphasizes the lack of regional epidemiological information and underscores the relevance of the present study for Central Asia. In contrast, studies from neighboring countries, such as Iran, have investigated the prevalence and genetic diversity of *S.* Abortusovis in aborted sheep (19), underscoring the knowledge gap that the present study aims to address. A study on the prevalence of *Salmonella* bacteria in South Africa shows that the most frequently detected serotype is *Salmonella Typhimurium (S. typhimurium)* (20). Information on *S. abortovis* in the United States is limited, as this pathogen is rarely reported and is not endemic to the region. According to experts from the World Health Organization (WHO), salmonellosis, as a zoonotic infection, is unparalleled in terms of the complexity of its epizootiological and epidemiological processes, as well as the challenges associated with its control (21). Modern outbreak tracing increasingly relies on molecular methods, with whole-genome sequencing (WGS) becoming standard (22–24).

In Kazakhstan, ovine salmonellosis has been reported for more than a decade, causing economic losses and lamb mortality (1). Isolated strains (*S.* Abortusovis AN 9/2 and 372) have been used as reference controls and for vaccine development (1, 25–27). Preventive measures require enhanced surveillance and sanitary–hygienic approaches (28). In Kazakhstan, sheep farming is predominantly based on extensive grazing systems with seasonal migrations. Large flocks often share pastures and watering points, which facilitates close animal contact and increases the risk of environmental contamination. Under such conditions, *S.* Abortusovis may persist in soil, water, and bedding, promoting horizontal transmission among susceptible ewes and thereby contributing to reproductive losses. Modern research requires not only traditional bacteriological diagnostics but also in-depth molecular analysis of isolated pathogen strains (29). However, molecular data on circulating *S.* Abortusovis strains in Central Asia are lacking.

To our knowledge, this study represents the first peer-reviewed report confirming the molecular identification of *Salmonella enterica* serovar Abortusovis from ovine abortion cases in Kazakhstan. These findings provide the first molecular evidence of this pathogen in the country and establish a reference point for future epidemiological surveillance and diagnostic assay development.

Therefore, the aim of this brief communication was to identify the circulation of *S.* Abortusovis in sheep in Kazakhstan and to confirm its species-level identity by 16S rRNA gene sequencing with a basic phylogenetic placement within the *S. enterica* complex; higher-resolution typing (e.g., MLST/WGS) was beyond the scope of this report.

## 2 Materials and methods

### 2.1 Sample collection and bacterial isolation

A total of 114 biological samples were examined during monitoring studies conducted between 2023 and 2025. *Salmonella* spp. were not detected in these samples, except for six *S.* Abortusovis isolates recovered from five aborted fetuses during the abortion outbreak in the Karaganda region in 2023. In addition, tissues and fluids from eight aborted sheep fetuses were collected during the outbreak between January and March 2023. Of these eight fetuses, five yielded positive cultures of *S.* Abortusovis, from which a total of six isolates were obtained. Of these cases, six *S.* Abortusovis isolates were recovered from five aborted fetuses during the single outbreak. Sample types included placenta, amniotic fluid, liver, spleen, and gastrointestinal content, as well as lung and kidney tissues from aborted fetuses and deceased lambs. Samples were collected aseptically and transported to the laboratory under refrigerated conditions (+4 to +8 °C) for further analysis. To exclude the presence of other common bacterial agents that may cause reproductive losses in sheep, including *Brucella* spp., *Listeria monocytogenes*, and *Campylobacter* spp., routine bacteriological examinations were also performed. No such pathogens were detected in the analyzed samples.

Initial bacterial enrichment was performed in meat-peptone broth (MPB) and incubated at 37 °C for 18–24 h. Subsequently, cultures were streaked onto meat-peptone agar (MPA) and MacConkey agar and incubated under the same conditions for up to 72 h to ensure optimal recovery of slow-growing *S.* Abortusovis colonies.

This procedure was conducted in accordance with the regional diagnostic protocol for ovine salmonellosis (KazSRVI, 2023), which is harmonized with the WOAH Manual of Diagnostic Tests and Vaccines for Terrestrial Animals (Chapter 3.10.3, *Salmonellosis*). Both guidelines recommend the use of non-selective or minimally selective media and extended incubation for slow-growing Salmonella serovars such as *S.* Abortusovis.

Colonies with morphology characteristic of *Salmonella* spp. were selected for further identification. Presumptive isolates were subjected to Gram staining and a biochemical test panel including glucose and lactose fermentation, urease activity, motility, hydrogen sulfide (H₂S) production, and lysine decarboxylase activity. Reference strain *S.* Abortusovis 372 was used as the comparator for biochemical and serological testing. This strain is the national working reference held in the Republic Collection of Microorganisms (RCM), previously curated at KazSRVI (2002–2015) and currently deposited at the National Research Center for Veterinary (NRCV, Astana), ensuring traceability, certified identity, and comparability with national diagnostic procedures.

### 2.2 Phenotypic and serological identification

Presumptive *Salmonella* isolates underwent Gram staining and a panel of biochemical tests, including glucose and lactose fermentation, urease activity, and motility. The isolates actively fermented glucose did not hydrolyze lactose or sucrose, and exhibited negative urease activity, which are characteristic features of the *Salmonella* genus. Serological confirmation was performed using agglutination with specific antisera (polyvalent and monoreceptor O- and H-Salmonella antisera, produced by the Saint Petersburg Research Institute of Vaccines and Sera and PETSAL, Russia) to identify *S.* Abortusovis. The obtained agglutination profile corresponded to the antigenic formula O: 4,12 (O group B); H: Phase 1 – c; Phase 2–1,6, consistent with the *S*. Abortusovis serotype according to the White–Kauffmann–Le Minor classification scheme. Morphological, cultural, biochemical, and serological properties were examined according to standard diagnostic protocols and Bergey’s Manual.

### 2.3 DNA extraction

Genomic DNA was extracted from pure bacterial cultures using the PureLink™ Genomic DNA Kit (Invitrogen, USA), following the manufacturer’s protocol. DNA integrity and concentration were assessed spectrophotometrically, and samples were stored at −20 °C until further use.

### 2.4 PCR amplification of the 16S rRNA gene

To confirm the genus-level identity of the isolated *Salmonella* strains, a fragment of the 16S ribosomal RNA gene was amplified using polymerase chain reaction (PCR). The reaction employed universal primers targeting conserved regions of the bacterial 16S rRNA gene: forward primer 16SF 190 (5′-ATTAGCTAGTTGGGGGTAAG-3′) and reverse primer 16SR 1,100 (5′-TTACGCGGATTCGACTTCA-3′).

PCR amplification was carried out in a total volume of 25 μL containing 2.5 μL of 10 × PCR buffer with MgCl₂ (Thermo Fisher Scientific, USA), 200 μM of each dNTP, 0.4 μM of each primer, 1 U of Taq DNA polymerase (Thermo Fisher Scientific, USA), approximately 50 ng of genomic DNA, and nuclease-free water to volume. Amplification was performed in a thermal cycler (Mastercycler Gradient, Eppendorf, Germany) under the following conditions: an initial denaturation at 94 °C for 3 min; 27 cycles of 94 °C for 30 s, annealing at 60 °C for 30 s, and 72 °C for 30 s; and a final elongation at 72 °C for 7 min.

The resulting PCR products were subjected to agarose gel electrophoresis (1.5%) in TAE buffer, stained with ethidium bromide, and visualized with a GelDoc Go imaging system (Bio-Rad, USA). The size of the amplified fragments was estimated by comparison with a 100 bp DNA ladder (Thermo Fisher Scientific, USA).

### 2.5 Purification and sanger sequencing

Amplicons were purified using Exonuclease I (Thermo Scientific) and Shrimp Alkaline Phosphatase (SibEnzyme, Russia). Sanger sequencing was conducted using the BigDye® Terminator v3.1 Cycle Sequencing Kit (Applied Biosystems) and analyzed on a 3,500 Genetic Analyzer (Applied Biosystems, USA).

### 2.6 Sequence assembly and phylogenetic analysis

Raw sequencing chromatograms were processed using Sequencing Analysis Software (Applied Biosystems) and assembled into consensus sequences using SeqMan (DNASTAR). Low-quality regions and primer sequences were trimmed. BLAST (NCBI) was used to compare the sequences against reference databases for taxonomic identification. Multiple sequence alignment and phylogenetic tree construction were performed in MEGA 11 using the Maximum Likelihood method. Evolutionary relationships were visualized to determine the placement of the studied strains within the *Salmonella* genus.

### 2.7 Ethical considerations

The study was approved by the Local Biological Ethics Committee of the Kazakh Research Veterinary Institute (Protocol #1, 14 July 2023). Informed consent was obtained from all sheep owners and participating veterinarians prior to blood sample collection.

## 3 Research results

### 3.1 Isolation of the pathogen using classical methods

A local isolate of *S.* Abortusovis (A) 58 was successfully obtained from pathological material collected during an ovine abortion outbreak in the Karaganda region. This isolate was one of the six *S.* Abortusovis isolates recovered during the same outbreak and was selected as a representative sample for detailed phenotypic and molecular characterization due to its high DNA quality and complete amplification profile. In total, six isolates of *S.* Abortusovis were obtained from five aborted fetuses during a single outbreak. The isolates originated from placenta (*n* = 2), amniotic fluid (*n* = 1), liver (*n* = 1), spleen (*n* = 1), and gastrointestinal content (*n* = 1). Strain (A) 58, which was selected for detailed characterization, was isolated from the placenta. All six isolates demonstrated identical morphological, biochemical, and serological characteristics, confirming that strain (A) 58 was fully representative of the group. Colonies grown on MPA exhibited morphology characteristic of *Salmonella* spp. The isolate was Gram-negative and demonstrated typical biochemical properties of *S.* Abortusovis.

Serological confirmation using polyvalent and monovalent antisera further identified the isolate as *S.* Abortusovis. Comparative analysis confirmed its equivalence to the reference strain 372. Pathological examination of the aborted fetuses revealed gross lesions consistent with *S.* Abortusovis infection, while culture confirmed the presence of the pathogen.

In addition to molecular identification, a comparative analysis of biological and biochemical properties was conducted for the field isolate *S.* Abortusovis (A) 58 and the reference strain *S.* Abortusovis 372. The evaluation included Gram staining, motility, growth characteristics on various culture media, biochemical reactions, and serological properties. Comparative analysis demonstrated that the field isolate (A) 58 and the pathogenic reference strain *S.* Abortusovis 372 exhibited identical growth, biochemical, and serological properties. Both strains fermented glucose, did not utilize lactose or sucrose, and were urease negative, consistent with the typical characteristics of *Salmonella* spp. No differences were observed in their reaction with specific antisera, confirming that strain (A) 58 dis-played the same phenotypic profile as the reference strain. No differences were observed in their reaction with specific antisera, confirming that strain (A) 58 displayed the same phenotypic profile as the reference strain. This comparative analysis was repeated three independent times, and the results were fully consistent across repetitions, supporting the reproducibility of the phenotypic identification. The isolate (A) 58 demonstrated agglutination patterns consistent with *S*. Abortusovis. The obtained antigenic profile corresponded to O antigens 4,12 (O group B) and H antigens Phase 1 – c; Phase 2–1,6, fully matching the *S*. Abortusovis serotype according to the White–Kauffmann–Le Minor classification scheme.

The detailed comparative phenotypic and serological characteristics of the field isolate *S.* Abortusovis (A) 58 and the reference strain *S.* Abortusovis 372 are summarized in Table 1.

**Table 1:** Comparative biological characteristics of the field isolate *S.* Abortusovis (A) 58 and the reference strain *S.* Abortusovis 372.

Biological properties	*S.* Abortusovis (A) 58	*S.* Abortusovis 372
Gram staining	−	−
Motility	+	+
Growth in MPB	Uniform turbidity	Uniform turbidity
Growth on MPA	S-form; small, round, convex, translucent colonies with smooth edges and glossy surface; grayish-blue in transmitted light	S-form; small, round, convex, translucent colonies with smooth edges and glossy surface; grayish-blue in transmitted light
Growth on Endo agar	Smooth, pale-pink, round transparent colonies	Smooth, pale-pink, round transparent colonies
Growth on bismuth sulfite agar	Small black colonies with metallic sheen; medium under colonies turns black	Small black colonies with metallic sheen; medium under colonies turns black
Growth on semi-solid agar	Motility observed after 24 h at 37 °C	Motility observed after 24 h at 37 °C
Catalase	+	+
Hydrogen sulfide production	+	+
Indole production	−	−
Methyl red test	+	+
Voges–Proskauer test	−	−
Sodium citrate utilization	+	+
Gelatin hydrolysis	−	−
Glucose	A + G	A + G
Lactose	−	−
Sucrose	−	−
Mannitol	A + G	A + G
Maltose	A + G	A + G
Dulcitol	A + G	A + G
Arabinose	A + G	A + G
Xylose	A + G	A + G
Fructose	A + G	A + G
Rhamnose	A + G	A + G
Physiological characteristics	Prototrophic	Prototrophic
Antigenic properties	Agglutination titer 1:1600; reacts with polyvalent and monoreceptor sera O-12,4; H-s (1st phase), 1,6 (2nd phase)	Agglutination titer 1:1600; reacts with polyvalent and monoreceptor sera O-12,4; H-s (1st phase), 1,6 (2nd phase)
Pathogenicity	Pathogenic to mice, pregnant ewes, and newborn lambs; lethal to mice at 150 CFU i.p.; abortogenic	Pathogenic to mice, pregnant ewes, and newborn lambs; lethal to mice at 150 CFU i.p.; abortogenic

A + G, formation of acid and gas; CFU, colony-forming units; i.p., intraperitoneally; “+”, positive; “-”, negative.

As presented in Table 1, both the field and reference strains exhibited identical cultural, morphological, biochemical, antigenic, and pathogenic profiles. The isolate (A) 58 demonstrated agglutination patterns and virulence characteristics consistent with *S.* Abortusovis, confirming its classification and suitability for further application in diagnostic assay development and vaccine production.

### 3.2 Molecular identification of the isolated strains

**Figure 2:**
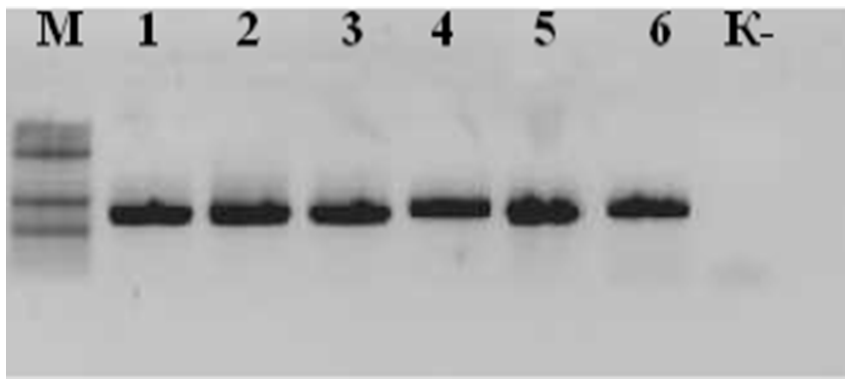
Agarose gel electrophoresis of PCR products targeting the 16S rRNA gene in six *Salmonella* isolates.

PCR amplification of the 16S rRNA gene fragment from six Salmonella isolates yielded a single specific amplicon of approximately 1,100 bp in all samples. Agarose gel electrophoresis revealed distinct, well-defined DNA bands with no nonspecific amplification products (Figure 2), confirming the specificity and efficiency of the selected primers. As the 16S rRNA gene is highly conserved among *Enterobacteriaceae*, this PCR assay was used to confirm genus-level affiliation with *Salmonella* spp., rather than serovar differentiation. Negative control lanes showed no amplification, further validating the absence of contamination during the PCR setup.

Lane M: DNA molecular weight marker (100 bp ladder, Thermo Scientific); Lanes 1–6: amplified 16S rRNA gene fragments (~1,100 bp); Lane K: negative control (no template DNA). All tested samples show specific amplification with no nonspecific products, confirming primer specificity and sample integrity.

As shown in Figure 2, all six analyzed *Salmonella* isolates exhibited amplification of specific DNA fragments approximately 1,100 bp in size as a result of polymerase chain reaction (PCR). The obtained PCR amplification results confirm the presence of *Salmonella*-specific sequences, demonstrating the high specificity of the applied method.

### 3.3 Nucleotide sequencing and quality assessment

The amplified PCR products were subjected to Sanger sequencing. Following sequence trimming and quality control, the final consensus sequences exceeded 650 bp. These sequences were submitted to the NCBI BLAST database for similarity analysis. All isolates demonstrated a 99% identity to multiple reference strains of *Salmonella enterica* subsp. *enterica*, including serovars Choleraesuis NR_074800.1 (accessed on February 2024), Paratyphi C NR_074899.1 (accessed on February 2024) and Paratyphi A NR_074935.1 (accessed on February 2024), as shown in Table 2. A full version of Table 2 is available in the Supplementary materials. Among these, the highest similarity was consistently observed with *Salmonella enterica* serovar Choleraesuis strain SC-B67, further supporting the classification of the isolates within *S. enterica* subsp. *enterica*.

**Table 2:** BLAST analysis of 16S rRNA gene sequences obtained from *Salmonella* isolates.

Strain	GenBank accession	Reference strain	% Identity
A58	NR_074800.1	*Salmonella enterica* subsp. *enterica* serovar Choleraesuis strain SC-B67	99%
A59	NR_074899.1	*Salmonella enterica* subsp. *enterica* serovar Paratyphi C strain RKS4594	99%
A60	NR_074935.1	*Salmonella enterica* subsp. *enterica* serovar Paratyphi A strain AKU12601	99%

The sequencing chromatogram shows clean, distinct peaks with no overlapping signals, indicating high sequence quality and the absence of contamination. The analyzed region corresponds to the conserved domain used for taxonomic identification.

### 3.4 Phylogenetic analysis

**Figure 3:**
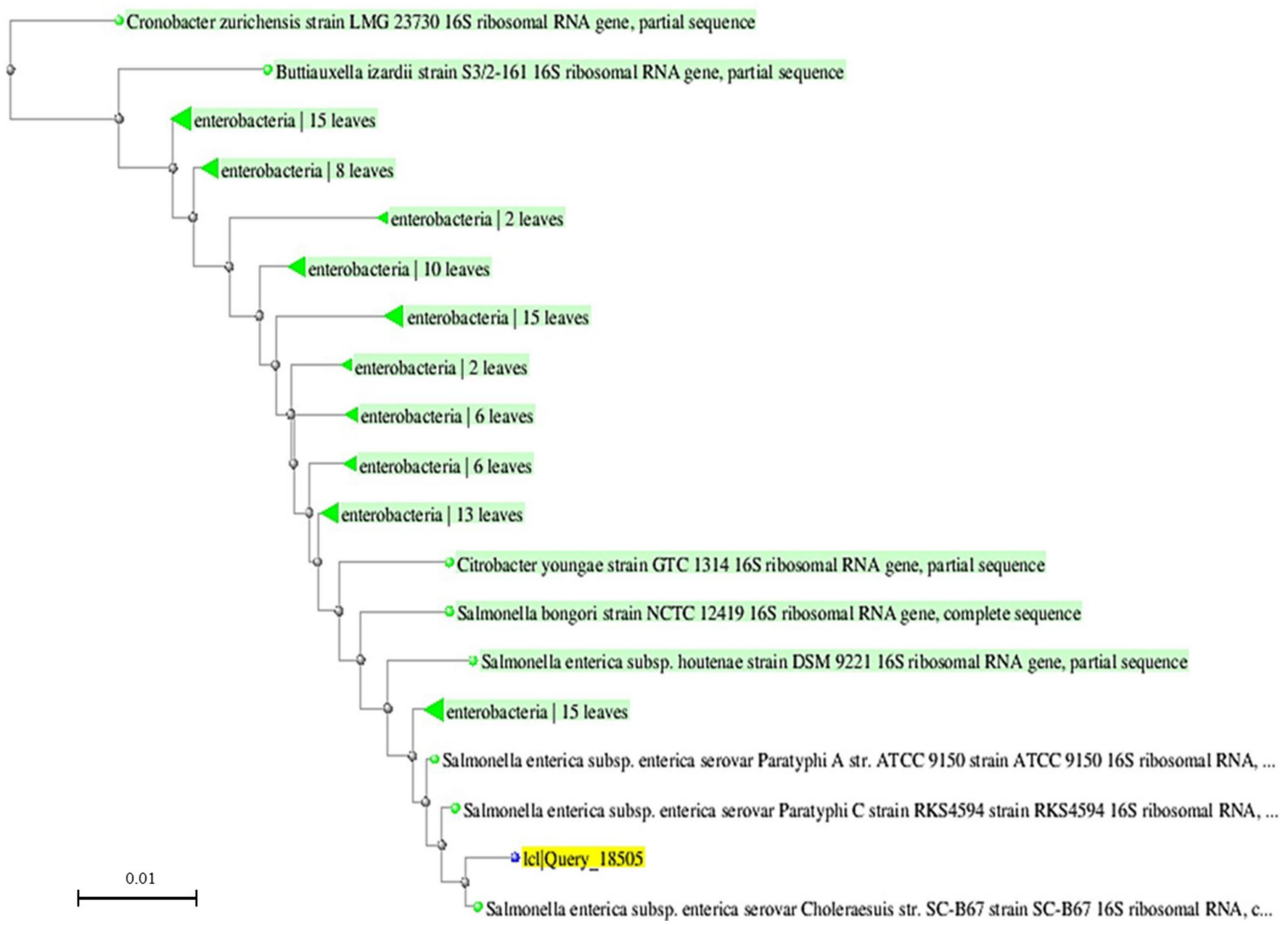
Phylogenetic tree based on 16S rRNA gene sequences of *Salmonella* isolates and reference strains.

To evaluate the evolutionary relationships of the isolated strains, a phylogenetic tree was constructed based on aligned 16S rRNA gene sequences using the Maximum Likeli-hood method implemented in MEGA 11 software. The resulting tree (Figure 3) demonstrated that the analyzed isolate (idQuery_18505) clustered closely with *S. enterica* serovar Choleraesuis and Paratyphi C reference strains, forming a well-supported monophyletic group within the *Salmonella enterica* subsp. *enterica* clade.

**Figure 4:**
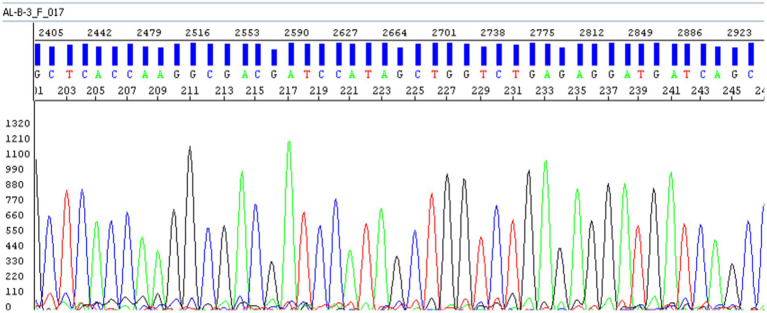
Electropherogram of the 16S rRNA gene nucleotide sequence from *Salmonella* isolate. This panel is a direct crop of the original sequencing trace display to remove interface margins only; no resampling, filtering, or per-peak editing was applied.

This close phylogenetic relationship reinforces the taxonomic identity established through BLAST analysis and supports the use of 16S rRNA sequencing as a reliable molecular marker for species-level identification of *Salmonella* spp. Taken together with classical bacteriology and serological typing (Sections 2.1–2.2; Results 3.1), gel-verified amplicons (Figure 2), clean Sanger chromatograms (Figure 4), and BLAST concordance (Table 1), these data support species-level identification; the 16S tree provides contextual placement only and does not imply serovar- or transmission-level inference.

Maximum-likelihood phylogeny (MEGA 11) of 16S rRNA gene sequences from the studied isolate and reference *Salmonella* taxa. Bootstrap support (1,000 replicates) is shown at the nodes. The studied isolate (idQuery_18505, highlighted in yellow) clusters within *S. enterica* subsp. *enterica*, in proximity to serovars Choleraesuis and Paratyphi C. Green labels denote clades within the *Enterobacteriaceae*, annotated by the number of leaves in each clade. The scale bar represents 0.01 substitutions per site. As expected for the conserved 16S locus, within-clade branch lengths are short, and serovar-level discrimination is not inferred. The reference sequences included in the analysis are indicated by their GenBank accession numbers, which provide information on their strain details and geographic origin available through the NCBI database.

As expected for a conserved 16S locus, within-clade branch lengths are short, and serovar-level discrimination is not inferred. Thus, the phylogenetic analysis results complement the BLAST-based identification, further confirming the classification of the studied isolate within *Salmonella enterica* subsp. *enterica*.

The phylogenetic analysis verified the close relationship of the examined strains with members of *Salmonella enterica* subsp. *enterica*, including serovars Paratyphi С and Choleraesuis, supporting the validity of their identification. The high nucleotide sequence identity of the 16S rRNA gene further confirms its role as a reliable genetic marker for bacterial taxonomic classification.

This study underscores the significance of molecular genetic methods for the rapid and precise identification of *Salmonella*, which can reduce diagnostic time, enhance result reliability, and optimize epizootiological monitoring processes.

## 4 Discussion

Our study successfully isolated *S.* Abortusovis from an ovine abortion case in Kazakhstan and confirmed its identity through both phenotypic methods and 16S rRNA gene sequencing. The isolates were found to be nearly identical (99%) to reference *Salmonella enterica* strains, supporting their classification within the *S. enterica* subsp. *enterica* lineage. Although the phylogenetic analysis based on 16S rRNA sequences supported the taxonomic placement of the isolates within *Salmonella enterica*, this approach has limited resolution for distinguishing among closely related serovars. We therefore acknowledge that 16S rRNA-based phylogeny provides genus- and species-level validation, but additional molecular markers (e.g., invA, fliC, fljB, or MLST loci) are required for definitive serovar differentiation.

To our knowledge, this is the first report from Kazakhstan documenting isolation and species-level identification by 16S, and biochemical identification proving that its belonging to *S.* Abortusovis from ovine abortion; we do not infer serovar-level diversity or transmission. A recent meta-analysis also indicated that *S.* Abortusovis isolates from small ruminants generally exhibit low levels of antimicrobial resistance, thereby posing limited zoonotic risk under slaughterhouse conditions.

The reliability of molecular diagnostics for *S.* Abortusovis largely depends on the DNA extraction strategy employed, as demonstrated by Belloy et al. (30). 16S rRNA sequencing has proved to be a reliable tool for the identification and taxonomic classification of *Salmonella*, offering faster and more cost-effective results than traditional culture-based methods. However, its highly conserved nature may not allow for sufficient discrimination between closely related serovars, as shown by the 99% identity of our isolates to multiple *S. enterica* serovars such as Choleraesuis and Paratyphi C. For more accurate differentiation, additional molecular markers or MLST are recommended. In agreement with this limitation, 16S rRNA sequencing alone cannot resolve *Salmonella* serotypes because of the gene’s high conservation across the genus. Therefore, in the present study, the serovar designation of the isolate as *S*. Abortusovis was based primarily on phenotypic and serological testing consistent with the Kauffmann–White–Le Minor classification scheme (O-12; 4; H-s). Although 16S rRNA sequencing provides genus-level confirmation, it is not sufficient for serovar-level identification due to its high conservation among *Enterobacteriaceae*. In future research, we plan to incorporate invA gene amplification, recommended by WOAH and ISO 6579-1:2017, along with the IS200-based conventional PCR described by Beuzón et al. (1997) to ensure molecular confirmation of *S*. Abortusovis and improve serovar differentiation. To achieve higher resolution in future work, we plan to perform molecular typing targeting the flagellin genes *fliC* and *fljB*, which encode phase 1 and phase 2 flagellar antigens, respectively. Sequencing these loci will provide confirmatory evidence for serotype assignment and facilitate comparative genomic analysis of *S.* Abortusovis isolates from Central Asia.

The availability of local isolates, such as strain (A) 58 obtained in this study, is of particular importance for vaccine development. Representative regional strains provide a relevant basis for the design of inactivated vaccines adapted to local epidemiological conditions. Similar approaches have been successfully applied in other regions where *S.* Abortusovis was implicated as a significant abortifacient pathogen (12).

The detection of *S.* Abortusovis in Kazakhstan aligns with sporadic reports of ovine reproductive losses across Central Asia, where bacterial abortion agents such as *Brucella melitensis* and *Listeria monocytogenes* remain endemic. The identification of *S.* Abortusovis emphasizes the need to integrate this pathogen into national abortion surveillance programs. Considering its host-adapted nature and limited zoonotic potential, local isolates may serve as reference strains for evaluating cross-protective immunity and for developing region-specific inactivated vaccines, as successfully implemented in Europe. Comparative genomic analyses with other ovine pathogens could further elucidate virulence mechanisms, gene conservation, and host adaptation traits critical for diagnostic assay refinement.

Taken together, the field isolate (A) 58 serves as a local reference for diagnostic assay development; any vaccine-related applications would require additional phenotypic and genomic characterization beyond the scope of this brief communication.

Overall, the results emphasize the importance of integrating molecular methods into routine veterinary diagnostics to enhance the surveillance of ovine salmonellosis in Kazakhstan. Generating molecular data on circulating *S.* Abortusovis strains strengthens diagnostic capacity and provides a scientific basis for improved prevention and control strategies in the region. Given the study design (single outbreak; small sample size) and use of a conserved marker, our inferences are intentionally narrow and do not address antimicrobial resistance, population structure, or transmission; future multi-site studies using MLST or WGS are warranted.

The primary advantage of this study is that it provides the first molecular evidence of *Salmonella enterica* serovar Abortusovis isolated from ovine abortion cases in Central Asia, thereby establishing a regional reference point for veterinary microbiology. The deposited isolates expand the national microbial collection and create opportunities for the development of region-specific diagnostic assays and preventive tools. At the same time, the scarcity of published data on the distribution and genetic variability of this pathogen in Central Asia highlights a critical gap in knowledge. Importantly, future investigations should prioritize systematic deposition of circulating *S.* Abortusovis isolates into international repositories and gene banks, ensuring their accessibility for comparative studies, validation of diagnostic systems, and long-term preservation as reference material. Such measures will contribute to a more comprehensive understanding of the epidemiology and evolutionary dynamics of this host-adapted pathogen in the region.

## 5 Conclusion

This study provides the first molecular confirmation of *Salmonella enterica* serovar Abortusovis circulation in sheep in Kazakhstan. The isolation and molecular identification of six field strains fill an important regional gap in knowledge of ovine salmonellosis. The obtained local isolates serve as valuable reference material for developing diagnostic assays, evaluating vaccine candidates, and strengthening epidemiological surveillance of reproductive infections in small ruminants. Future genomic studies and coordinated monitoring will be essential to trace transmission pathways and guide effective control strategies across Central Asia.

## Data Availability

The authors selected the following statement: The original contributions presented in the study are included in the article/Supplementary material, further inquiries can be directed to the corresponding author.
