# Supplementary material for: First report on identification of Salmonella Abortusovis from ovine abortion cases in Kazakhstan
Source: Front Vet Sci. 2026 Jan 19;12:1717314. doi: 10.3389/fvets.2025.1717314 (PMC12862935; doi:10.3389/fvets.2025.1717314)
Supplement: Supplementary file 1 [file Supplementary_file_1.docx]

**Table 2 - BLAST analysis of 16S rRNA gene sequences obtained from Salmonella isolates**

| Strain Name | 16S rRNA Gene Fragment Sequence | Identification of Nucleotide Sequences in the International Database (NCBI BLAST) | | |
| --- | --- | --- | --- | --- |
|  |  | GenBank Accession Number or Strain Collection Number | Strain Name | % Identity |
| *Salmonella abortusovis* | GGAGGGGGTACTACTGGAACGGTGGCTAATACCGCATAACGTCGCAAGACCAAAGAGGGGGACCTTCGGGCCTCTTGCCATCAGATGTGCCCAGATGGGATTAGCTTGTTGGTGAGGTAACGGCTCACCAAGGCGACGATCCCTAGCTGGTCTGAGAGGATGACCAGCCACACTGGAACTGAGACACGGTCCAGACTCCTACGGGAGGCAGCAGTGGGGAATATTGCACAATGGGCGCAAGCCTGATGCAGCCATGCCGCGTGTATGAAGAAGGCCTTCGGGTTGTAAAGTACTTTCAGCGGGGAGGAAGGTGTTGTGGTTAATAACCACAGCAATTGACGTTACCCGCAGAAGAAGCACCGGCTAACTCCGTGCCAGCAGCCGCGGTAATACGGAGGGTGCAAGCGTTAATCGGAATTTACTGGGCGTAAAGCGCACGCAGGCGGTCTGTCAAGTCGGATGTGAAATCCCCGGGCTCAACTGGGAACTGCATTCGAAACTGGCAGGCTTGAGTCTTGTAGAGGGGGGTGGAATTCCAGGTGTAGCGGTGAAATGCGTAGAGATCTGGAGGAATACCGGTGGCGAAGGCGGCCCCTGGACAAAGACTGACGCTCAGGTGCGAAAGCGTGGGGAGCAACAGGATTAGATACCCTGGTAGTCCACGCCGTAAACGATGTCTACTTGGAGGTTGTGCCCTTGAGGCGTGGCTTCCGGAGCTAACGCGTTAAGTAGACCGCCTGGGGAGTACGGCCGCAAGGTTAAAACTCAAATGAATTGACGGGGGCCCGCACAAGCGGTGGAGCATGTGGTTTAATTCGATGCAACGCGAAGAACCTTACCTGGTCTTGACATCCACGGAAGTTTTCAGAGATGAGAATGTGCCTTCGGGAACCGTGAGACAGGTGCTGCATGGCTGTCGTCAGCTCGTGTTGTGAAATGTTGGGTTAAGTCCCGCAACGAGCGCAACCCTTATCCTTTGTTGCCAGCGATTAGGTCGGGAACTCAAAGGAGACTGCCAGTGATAAACTGGAGGAAGGTGGGGATGACGTCAAGTCATCATGGCCCTTACGACCAGGGCTACACACGTGCTACAATGGCGCATACAAAGAGAAGCGAGCTCGCGAGAGCAAGCGGACCTCATAAAGTCCGTCGTAGTCC | [NR_074800.1](http://www.ncbi.nlm.nih.gov/nucleotide/444439485?report=genbank&log$=nucltop&blast_rank=1&RID=0BJURVAJ01R) | [Salmonella enterica subsp. enterica serovar Choleraesuis str. SC-B67 strain SC-B67](http://blast.ncbi.nlm.nih.gov/Blast.cgi#alnHdr_444439485) | 99% |
|  |  | [NR_074899.1](http://www.ncbi.nlm.nih.gov/nucleotide/444439584?report=genbank&log$=nucltop&blast_rank=2&RID=0BJURVAJ01R) | [Salmonella enterica subsp. enterica serovar Paratyphi C strain RKS4594 strain RKS4594](http://blast.ncbi.nlm.nih.gov/Blast.cgi#alnHdr_444439584) | 99% |
|  |  | [NR_074935.1](http://www.ncbi.nlm.nih.gov/nucleotide/444439620?report=genbank&log$=nucltop&blast_rank=3&RID=0BJURVAJ01R) | [Salmonella enterica subsp. enterica serovar Paratyphi A str. AKU_12601 strain AKU12601](http://blast.ncbi.nlm.nih.gov/Blast.cgi#alnHdr_444439620) | 99% |
